# Er,Cr:YSGG 2780 nm Laser Treatment of Lip Melanin Hyperpigmentation

**DOI:** 10.1155/2021/6621341

**Published:** 2021-07-15

**Authors:** Manaf Taher Agha, Pavel Polenik, Mawada Hassan

**Affiliations:** ^1^Charles University in Prague, Department of Stomatology (Pilsen), Adjunct Faculty Member Ajman University Faculty of Dentistry, Ajman, UAE; ^2^Charles University in Prague, Department of Stomatology (Pilsen), Prague, Czech Republic; ^3^Ajman University Faculty of Dentistry, Ajman, UAE

## Abstract

**Background:**

In the current research, Er,Cr:YSGG laser been used in ablative mode to peel the pigmentation, and many sessions were done to completely remove the melanin pigmentations.

**Materials and Methods:**

85 patients were selected and Er,Cr : YSGG laser with 2780 nm wavelength in ablative mode (iPlus, Biolase, USA) was used to treat patients with dark lip melanin pigmentation, using gold handpiece and MZ 10 tip (diameter of 1 mm), and the parameters were set as follows: energy density 28.7 J/cm^2^, frequency 30 Hz, water cooling 100%, and air cooling 60%.

**Results:**

The vast majority of the patients (82.4%) had mild adverse effects after the lip depigmentation and 74.1% of patients reported complete improvement of the lip with a high satisfaction rate (84.7%) during the follow-up period with a low level of relapse.

**Conclusion:**

Er,Cr:YSGG 2670 nm is an effective tool to achieve excellent esthetic results in the treatment of lip melanin pigmentation; it is very well tolerated by patients with minimal adverse effects.

## 1. Background

Lips are an important part of our smiles; the shape and color are crucial elements of the lip's beauty.

The color of the lips is generally pink and reddish but normally varies from person to person depending on factors such as melanin production, skin color, vascularity, and dryness (dehydration) of the lips.

Lips color changes may also be influenced by the following factors [1–8]:

Sun exposure
Smoking
Vascular lesions: hemangioma, hematoma, and venous lake
Anemia: causing pale lips
Syndromes and diseases: Peutz–Jeghers' syndrome, Albright syndrome, and Addison's disease
Medications: antimalarials: clofazimine, chloroquine, and amodiaquine
    Antimicrobial agents: minocycline
    Antiretroviral agents: zidovudine
    Antifungals: ketoconazole
Topical chemicals: cosmetic products and lipstick
Hormones: melanocyte-stimulating hormone and adrenocorticotropic hormone
Malignant pigmented lesions
Inflammations
Vascularity
Skin color (genetic)

These changes in the color of the lips are usually considered unaesthetic and can affect the patient's social activities and self-confidence and patients are seeking solutions.

Many techniques have been used for the treatment of lip pigmentation depending on the causes of these pigmentations [8].

Melanin pigmentation normally exists in the epithelial layer and might reach the basal layer, making these pigmentations superficial and easy to reach and eliminate.

Lip melanin depigmentation techniques may include the following [9, 10]:

  Laser therapy
  Cryotherapy
  Pink tattoo
  Photodynamic therapy
  Chemical peeling (microabrasion)
  Topical medicated agents (Vit. C, arbutin, hydroquinone, and liquid nitrogen)

All the previous treatments results' have different outcomes and adverse effects and esthetic results might not be satisfactory for the patients; however, lasers achieved good acceptable results esthetically and are tolerated by patients [11, 12].

Like the rest of the skin, lips have layers (epidermis, dermis, and hypodermis), although the difference in the lips is that the stratum corneum is far thinner than it is anywhere else, which is why vascularity may change the color of vermilion easily.

Lips are very rich with nerve endings, which make them very sensitive to pain, and have minor salivary glands; hence, any procedures done on the lips should take this anatomy into consideration to avoid scars, mucoceles, and pain [13].

Understanding the structure of the lip (thickness, anatomy, and sensitivity due to rich innervation and superficial nerve endings) and the thermal impact of the different wavelengths is important to avoid any wavelength that might penetrate deeply and cause an undesired thermal effect. Erbium lasers are the least penetrating compared to the near-infrared and visible wavelengths, which make them a good choice for such procedures.

Using near-infrared lasers like diodes 810 nm, 940 nm, and 980 nm and Nd : YAG is likely to cause an undesired thermal impact. *Q*-switched 532 nm is less penetrating than near-infrared; however, still, the thermal influence could irritate the lips, and blue diode laser 445–450 nm is even less penetrating [14].

Published papers and case reports on laser treatment for lip melanin pigmentation are very few, and some are not reliable due to the very low number of samples or case reports, some cases are done with near-infrared laser with the lips swelling and a painful experience for the patient, and some cases were observed in our clinic with scars or with a prolonged healing period, which could extend 4–6 weeks [11, 12, 15–17].

Erbium lasers have been used in fractional mode by using a fractional handpiece that distributes the laser beam in multiple points to eliminate the pigmentation, help with skin rejuvenation, and reduce thermal impact with acceptable results and patient comfort [18].

In this research, a Er,Cr:YSGG laser has been used in the ablative mode to peel the pigmentation, and many sessions were provided to completely remove the melanin pigmentations.

## 2. Materials and Methods

**Figure 1:**
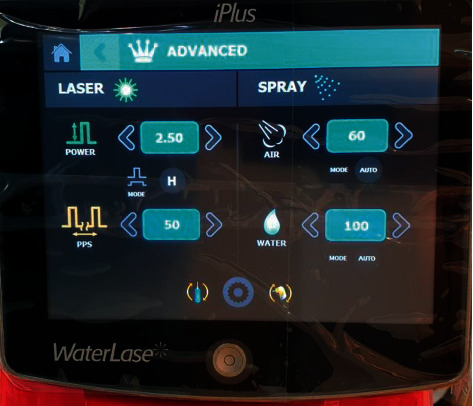
The settings showing on the screen of the Er Cr : YSGG laser machine.

**Figure 2:**
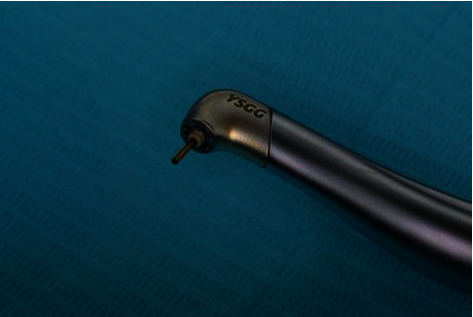
Gold handpiece with MZ10 tip.

(A) Er,Cr : YSGG laser with 2780 nm wavelength in ablative mode (iPlus, Biolase, USA) was used to treat patients with dark lip melanin pigmentation, using a gold handpiece and MZ 10 tip (diameter of 1 mm), and the parameters were set as follows:
Energy density 28.7 J/Cm^2^, frequency 30 Hz, water cooling 100%, and air cooling 60% are listed in Figures 1 and 2.
(B) 85 patients (84 females, 1 male) were selected and diagnosed; all cases were healthy patients with no other systemic diseases, aged 20–48 years old, with melanin hyperpigmented lips. The lips pigmentation underwent assessments according to the following index:
    Score 0: normal red/pink lips without any islands of dark pigmentation.
    Score 1: dark small isolated islands/dots of pigmentation
    Score 2: dark big islands of pigmentation
    Score 3: dark pigmented hued line that contour the lip
    Score 4: dark wide strip that covers the whole vermilion of the lip
    Score 5: dark wide strip that includes the vermilion and mucosal side of the lip
(C) According to the previous index, the distribution of the cases was as follows:
    Two patients scored 1.
    Four patients scored 2.
    Thirty-one patients scored 3.
    Forty-eight patients scored 4.
(D) All cases went through 3–8 sessions according to the score of pigmentation; topical anesthetic cream 2.5% lidocaine/prilocaine (EMLA) cream has been applied 20 minutes prior to each session.

### 2.1. Outcome Measurements

1. Clinical parameters: Adverse effects and postop pain were assessed between sessions. Healing time and color improvements were assessed after 2 weeks of the final session.
2. Esthetic parameters: photographs of smiles were taken before and after treatments, and then they were assessed by three calibrated professionals: 2 dermatologists and 1 dentist; the procedure was blinded and randomized and repeated for validity and repeatability. Ratings were classified on a five-point smile improvement scale.
3. Satisfaction has been evaluated after 3 months of treatment.
4. Relapse has been evaluated until 18 months.

### 2.2. Indices of Measurements

**Figure 3:**
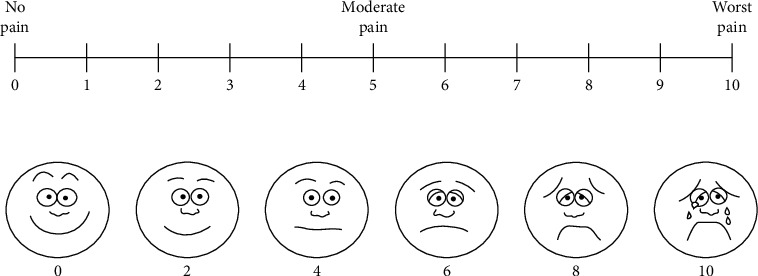
VAS index to evaluate pain.

(1) Adverse effects include scares, herpes labialis, severe crust, bleeding, and swelling:
  (0) No adverse effects
  (1) Mild adverse effects
  (2) Moderate adverse effects
  (3) Severe adverse effects
(2) Wound healing was scored (14 days after final session) on a 2-scale index:
  1. Complete healing
  2. Partial healing (incomplete epithelial formation)
(3) Pain after the procedure was scored using the VAS index of pain measurement:
  1. No pain ascending to 10–severe disabling pain (Figure 3)
(4) Color improvement objective recording of lip color variation was assessed using a five-point smile improvement scale:
  1. No improvement
  2. Little improvement
  3. Average improvement
  4. Great improvement
  5. Complete improvement (natural lip color)
(5) Satisfaction with treatment was documented depending on the patient's opinion, measured on five points scale after 3 months of treatment:
  1. Very dissatisfied
  2. Dissatisfied
  3. Neither satisfied nor dissatisfied
  4. Satisfied
  5. Very satisfied
(6) Relapse was measured through a period of 18 months:
  1. No relapse = no pigmentation at all
  2. Mild relapse = mild pigmentation (separated islands)
  3. Medium relapse = pigmentation is obvious and forming a small strip
  4. Severe relapse = pigmentation is obvious and forming a wide strip

### 2.3. Statistical Analysis

The data collected were tabulated and the statistical analyses were achieved using SPSS 26.0 (SPSS, Inc, Chicago, Illinois, United States).

The categorical variables of the present study were organized and tabulated as descriptive results (frequencies and percentages).

For the inferential statistics, the assumption of normality was established to check the validity of the parametric test using the Shapiro–Wilk test. Significance in the Shapiro–Wilk test revealed *p* < 0.05; as a result, we reject the null hypothesis and the data were not normally distributed as the data were categorical. Consequently, nonparametric one-sample chi-square (X^2^) test and one-sample binomial test were used to test our hypothesis, in which null hypothesis (H0) assumes that treatment outcomes of dark lip using Er,Cr:YSGG laser include mainly no adverse effect, complete healing, equal probabilities of pain score after the procedure, complete improvement of the lip color using a five-point smile improvement scale with high satisfaction, and no relapse during the follow-up period, In contrast, alternative hypothesis (H1 ≠ H0) states there is a difference between the result and our hypothesized claim in relation to different parameters (adverse effect, healing, pain, satisfaction, color improvement, and relapse).

In order to ascertain the relation between different parameters, Spearman's correlation was selected to compare the strength of the effect of different outcomes in relation to each other.

Moreover, one-way ANOVA (summary data using Syntax command in SPSS and Kruskal–Wallis H test were used to compare our results with those of another study in which they used Q-switched Nd : YAG 1046 nm and Q-switched Nd : YAG 532 nm lasers.

The statistical significance (*p* value) was set in the current study at below 0.05 and 95% confidence level.

## 3. Results

**Figure 4:**
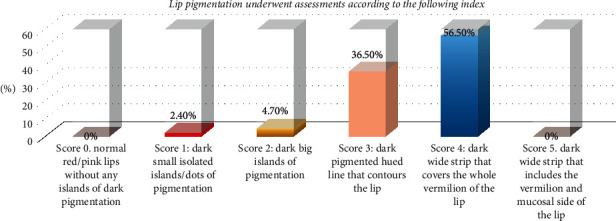
Cases distribution according to the melanin pigmentation assessment.

The distribution of the cases as demonstrated in Figure 4, which showed 48 (56.5%) of the involved cases scored 4 as baseline before the procedure.

All outcomes collected from 85 patients involved in the current study are tabulated and summarized in Table 1.

1. Null hypothesis:
2. *H*_0_ = treatment outcomes of dark lip include mainly no adverse effect, complete healing, equal chances of pain score after the procedure, complete improvement of the lip color using a five-point smile improvement scale with high satisfaction, and no relapse during the follow-up period.
3. Alternative hypothesis:
4. H_1_ ≠ H_0_: there is a difference between the result and our hypothesized claim.

### 3.1. Adverse Effects Including Scares, Herpes Labialis (Reactivation), Severe Crust, Bleeding, and Swelling

**Figure 5:**
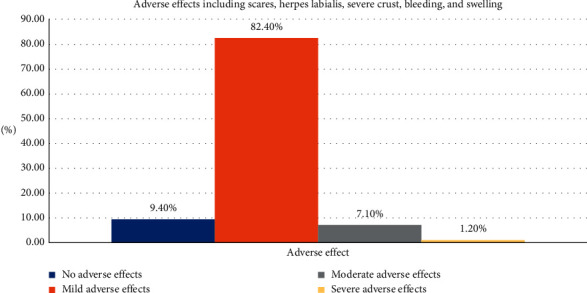
The results of adverse effects including scares, herpes labialis, severe crust, bleeding, and swelling.

Observation of the adverse effects when using Er,Cr:YSGG laser revealed that the vast majority of the patients (82.4%) had mild adverse effects after the lip depigmentation. Moreover, patients treated with Er,Cr:YSGG laser experience moderate or severe adverse effects after the surgery only (7.1% and 1.2%, respectively) and few patients (9.4%) had no adverse effect after the procedure; thus, significant difference was found and H_0_ was rejected (*p*=0.001), as shown in Table 2 and Figure 5.

### 3.2. Wound Healing Scored 14 Days after Treatment on a 2-Scale Index

**Figure 6:**
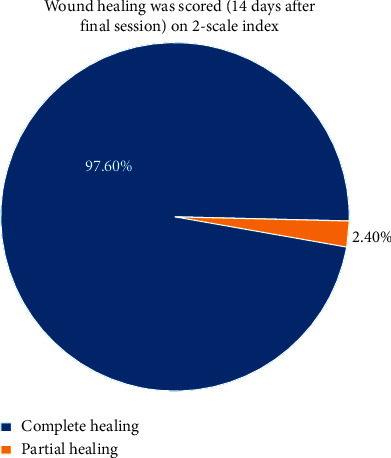
Results of wound healing was scored (14 days after final session) on a 2-scale index.

Generally, most of the patients had complete wound healing 14 days after the procedure (97.6%) when using Er,Cr:YSGG laser with no significant difference in respect to the null hypothesis (*p*=0.239), as shown in Table 2 and Figure 6.

### 3.3. Pain (Burning Sensation) after the Procedure Was Scored Using VAS Index of Pain Measurement

**Figure 7:**
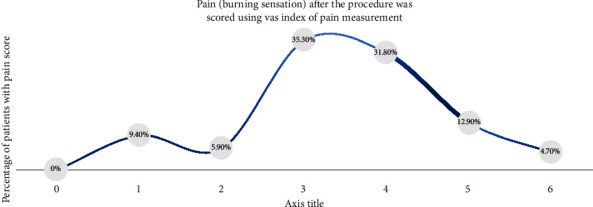
The pain (burning sensation) scores taken between the recorded sessions using Er,Cr : YSGG laser.

VAS index of pain measurement was selected and scores from each patient werereported after the treatment; consequently, 35.3% had score 3 of pain after the treatment using Er,Cr:YSGG laser, 31.8% scored 4, and 12.9% scored 5. Then, 9.4%, 5.9%, and 4.7% of patients gave a score of 1, 2, and 6, respectively, with statistically significant differences (*p*=0.001) between different patients in relation to the null hypothesis of equal probabilities between them, as shown in Table 2 and Figure 7.

### 3.4. Color Improvement Objective Recording of Lip Color Variation Using a Five-Point Smile Improvement Scale

**Figure 8:**
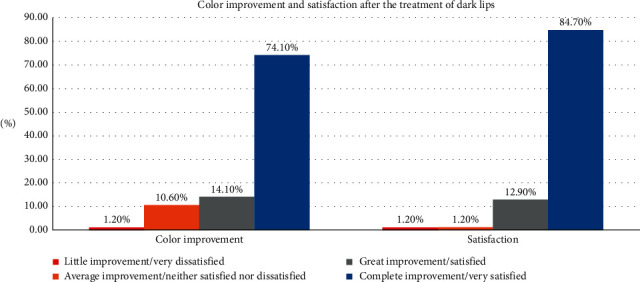
Color improvement and satisfaction after the treatment of dark lips after Er,Cr : YSGG laser treatment.

The current study also raises the issue of color improvement using ER,Cr: YSGG laser, in which 74.1% of patients reported complete improvement of lip compared to only 14.1% with great improvement followed by 10.6% with average improvement and only 1.2% with little improvement. One-sample *X*^2^ test showed unequal distribution of the patients with equal probabilities (*p*=0.001), yet there is no significant difference between the result (*p*=0.110) and the null hypothesis (complete improvement is expected in most of the cases), as shown in Table 2 and Figure 8.

### 3.5. Satisfaction with Treatment Was Documented Depending on the Patient's Opinion

Detailed determination of the successful of Er,Cr:YSGG laser was studied by mean of measuring the satisfaction of all patients involved in the study after 6 months, which revealed a high rate of satisfaction when using Er,Cr:YSGG laser (84.7%). On the other hand, only 1.2% of patients were not satisfied with the treatment, giving a significantly low level (*p*=0.513) when compared the result to our null hypothesis, as shown in Table 2 and Figure 8.

### 3.6. Relapse Measured after 18 Months of “Photo Documentation”

**Figure 9:**
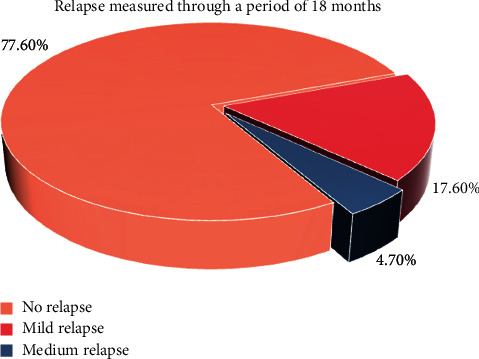
The relapse scores measured 18 months after the final session.

One of the most important aspects measured in the current prospective study is the relapse of the treatment after 18-month follow-up, resulting in 66 cases (77.6%) with no relapse, followed by 15 cases (17.6%) of mild relapse after using Er,Cr:YSGG laser, while only 4 cases were reported with medium relapse (4.7%) with significant difference between them in terms of relapse (*p*=0.001), as shown in Table 2 and Figure 9.

### 3.7. Spearman's Correlation between Different Outcomes of Lip Depigmentation Using Er,Cr:YSGG Laser

Spearman's correlation analysis was used to study the strength between variables in relation to each other (adverse effect, pain, healing, color improvement, satisfaction, and relapse). There was a strong positive correlation in relation to satisfaction (*r* = 0.790, *p*=0.001) and color improvement; thus, the satisfaction increases with the complete improvement of the lip color after the treatment.

Correlation analysis also showed a strong negative correlation in relation to satisfaction (*r* = −0.739, *p*=0.001) in addition to color improvement (*r* = −0.908, *p*=0.001) with the relapse outcome; accordingly, satisfaction and color improvement had an inverse proportion to the relapse, indicating that more color improvement results in less relapse over a short period of time.

Similarly, adverse effect (*r* = −0.367, *p*=0.001) revealed a weak negative correlation (inverse proportion) in relation to wound healing (Table 3).

## 4. Discussion

It is known that when using a laser, there is some collateral thermal impact that should always be minimized, when using a deep penetrating wavelength especially on very thin sensitive skin, such as lips. This led us to use the Er,Cr:YSGG as the penetration of this wavelength is superficial and with a less thermal impact with the ability to use water and air cooling from the machine.

Many articles have been published regarding lip melanin depigmentation with a laser using near-infrared (1064, 940, and 810 nm) and also the *Q*-switched 532 nm. Most of the previous studies mentioned adverse effects such as reactivation of herpes labialis, dark crust, scars, swelling, dark pigmentation, burning sensation, patient discomfort, and pain [11, 12, 15–17].

**Figure 10:**
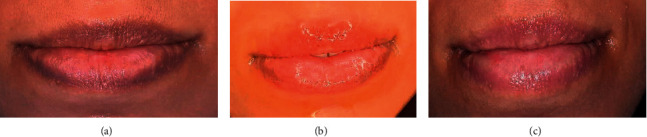
(a) Before, (b) mild swelling after the session, and (c) after complete healing.

**Figure 11:**
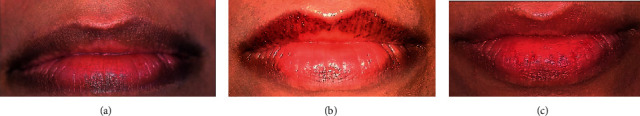
(a) Before, (b) mild crust, and (c) after healing.

**Figure 12:**
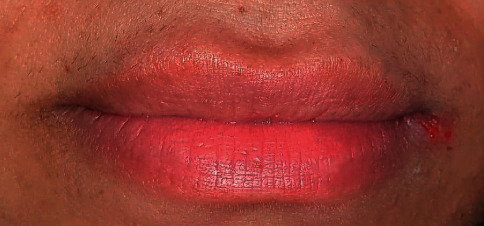
Reactivated herpes labialis.

According to our results, the adverse effect in this study is that the majority of the cases experienced mild adverse effect, which was mild transient swelling (Figure 10) for the first few hours after the sessions resolving between 4 and 12 hours, followed by mild crust (Figure 11) that will peel away in 3–4 days. However, those effects were highly tolerable by the patients. It should be mentioned that we have encountered 2 cases of reactive herpes labials (Figure 12) and one case of Vit. E localized allergy as we asked the patients to apply Vit. E on lips after the sessions. One case of severe adverse effect was registered with a deep color crust combined with a burning sensation that lasted for a week, but the case healed well with a longer time compared with the other cases.

A case report using 940 nm showed severe swelling with a dark pigmented crust and scars and the scar was still showing in the one-month follow-up photo, which we believe happened due to the thermal impact of the 940 nm [15].

**Figure 13:**
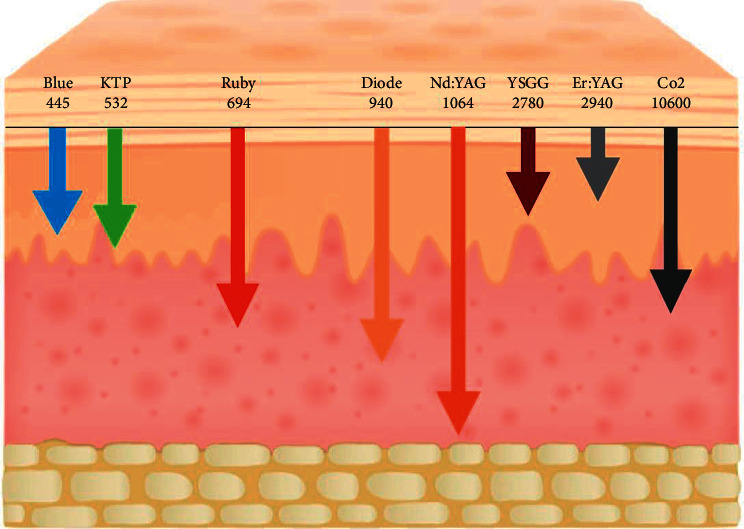
Penetration depth of different laser wavelengths.

As expected, most of the cases in this study healed within the 14 days after the last session, which is a logical result when using Er,Cr:YSGG wavelength that will not penetrate deeply in addition to water and air cooling, which will reduce thermal impact significantly on the lip tissue leading to faster healing. The penetration of different wavelengths is demonstrated in Figure 13.

When patients are asked to describe the pain and score using the VAS index (Figure 4), where 0 is no pain and 10 is the worst unbearable pain, they all preferred to describe it as a burning sensation and not pain.

More than 82% scored 4 and less, which is a mild and very bearable burning sensation; other studies showed different levels of burning sensation scored [11, 12, 15–17].

**Figure 14:**
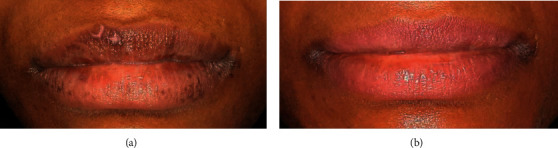
Upper and lower lips hyperpigmentation and scars due to a previous attempt as the patient claimed. (a) Before and (b) after the scars correction.

The results of the color improvement showed that more than 88% had great-to-excellent complete improvement, with restoring the pink color and the shape with no scars or any undesirable esthetic results. In Figures 14(a) and 14(b), the ability to remove the scars in addition to removing the melanin pigmentation by resurfacing the lip skin is noted.

The satisfaction of the patients was very high as more than 97% were satisfied or even very satisfied with the esthetic results and these results are related to the color improvement in the cases.

**Figure 15:**
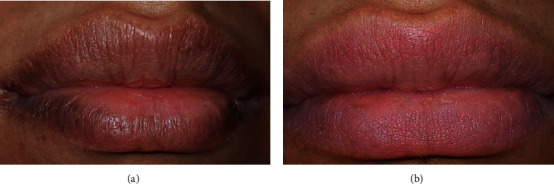
(a) Before and (b) 14 days after the last session.

One of the very satisfied cases (Figure 15) is where the patient claimed that she had tried many techniques before but was not satisfied with any one of them.

Comparing our results with a study by Limpjaroenviriyakul et al. in [12], using Q-switched 1064 and 532 nm laser in Bangkok on 30 cases, as shown in (Table 4), the percentage of the color improvement with Er,Cr:YSGG is higher than both of the Q-switched 1064 and 532 nm, and the satisfaction was also higher when using Er,Cr:YSGG.

Relapse is possible in this type of melanin pigmentation [11, 12] as it depends on the activity of the melanocytes that produce the melanin, especially in some areas of the world due to genetic (racial) factors and other factors like sun exposure and smoking.

The results of this study showed more than 77% of no relapse within the 18-month follow-up period, 17.6% of the cases with mild relapse, and only 4.7% with medium relapse. On the other hand, photo documentation for relapse after two years on the study done by the same authors Agha and Polenik in 2020 [19] revealed that relapse mainly occurs with Er,Cr:YSGG laser compared to the diode laser with no statistically significant difference as diodes are well absorbed in the melanin pigmentation and the diodes are more penetrating in the tissue depth, which probably reduces the possible presence of nonpeeled pigmented cells or particles. Furthermore, patients in the current study were asked to avoid sun exposure and use lip balm with sun protection to reduce the possibility of activating the melanocytes. Smoker patients were asked to quit smoking for the same reasons.

## 5. Conclusion

  Er,Cr:YSGG 2670 nm is an effective tool to achieve excellent esthetic results in the treatment of lip melanin pigmentation and it is very well tolerated by patients with minimal adverse effects.
  The follow-up period needs to be longer to evaluate the relapse, even though within this period, relapse was very minimal.
  It seems that erbium wavelengths can achieve a better esthetic result with minimal adverse effects compared with other wavelengths, but there should be comparison studies in the future to evaluate many wavelengths and their results.

## Figures and Tables

**Table 1:** Descriptive analysis of the current study using Er,Cr : YSGG laser.

Outcomes after treatment^*∗*^	Frequency (*n* = 85)	Percentage (%)
Adverse effects include scares, herpes labialis (reactivation), severe crust, bleeding, and swelling		
No adverse effects	8	9.4
Mild adverse effects	70	82.4
Moderate adverse effects	6	7.1
Severe adverse effects	1	1.2

Wound healing was scored (14 days after final session) on a 2-scale index		
Complete healing	83	97.6
Partial healing	2	2.4

Pain after the procedure was scored using VAS index of pain measurement		
Score 1	8	9.4
Score 2	5	5.9
Score 3	30	35.3
Score 4	27	31.8
Score 5	11	12.9
Score 6	4	4.7

Color improvement objective recording of lip color variation using a smile improvement scale		
Little improvement	1	1.2
Average improvement	9	10.6
Great improvement	12	14.1
Complete improvement	63	74.1

Satisfaction with treatment depending on the patient's opinion		
Very dissatisfied	1	1.2
Neither satisfied nor dissatisfied	1	1.2
Satisfied	11	12.9
Very satisfied	72	84.7

Relapse measured through a period of 18 months		
No relapse	66	77.6
Mild relapse	15	17.6
Medium relapse	4	4.7

^*∗*^One-sample chi-square test showed unequal distributions of all the outcomes within each category (*p*=0.001).

**Table 2:** Statistical analysis using the one-sample chi-square test (*X*^2^) and one-sample binomial test to determine the significant difference between our null hypothesis to the actual observed data using Er,Cr:YSGG laser.

	Adverse effect	Wound healing after 14 days	Pain a using VAS index	Color improvement	Satisfaction after 3 months	Relapse after 18 months
*X* ^2^	145.078	^*∗∗*^	45.941	1.875	0.398	9.326
Test statistics	208.843	2.000	45.941	7.547	3.277	19.280
Df	3	1	5	4	4	3
*p* value^*∗*^	0.001	0.239	0.001	0.110	0.513	0.001

^*∗*^The distribution of the categories was reported with the specified probabilities according to the null hypothesis. ^*∗∗*^One-sample binomial test was performed.

**Table 3:** Correlation between different outcomes evaluated during Er,Cr : YSGG laser thearpy.

			Satisfaction	Adverse effects	Wound healing	Pain score using VAS index	Relapse after 18 months
Spearman's rho correlation	Color improvement	Correlation coefficient	0.790	−0.026	0.326	0.704	−0.908
		Sig.	0.001	0.408	0.001	0.001	0.001
	Satisfaction	Correlation coefficient		−0.106	0.420	0.650	−0.739
		Sig.		0.168	0.001	0.001	0.001
	Adverse effects	Correlation coefficient	−0.106		−0.367	−0.046	−0.053
		Sig.	0.168		0.001	0.338	0.313
	Wound healing	Correlation coefficient	0.420	−0.367		−0.254	−0.270
		Sig.	0.001	0.001		0.010	0.006
	Pain score using VAS index	Correlation coefficient	0.650	−0.046	−0.254		−0.646
		Sig.	0.001	0.338	0.010		0.001

**Table 4:** Comparing Er, Cr:YSGG to Q-switched Nd : YAG 1064 nm laser and Q-switched Nd : YAG 532 nm laser in the treatment of hyperpigmented lips (comparison between different studies).

	Er,Cr:YSGG	Q-switched Nd : YAG 1064-nm	Q-switched Nd : YAG 532-nm	*p* value^*∗*^
Color improvement	74.1%^A^	5%^B^	40.17%^A^	0.001
Satisfaction	3.80 (0.57)^A^	0.53 (0.52)^B^	1.73 (0.46)^C^	0.001

^*∗*^Kruskal–Wallis test was used for the color improvement (test statistics = 33.36), One-way ANOVA for the satisfaction and pain, Df = 2.

## Data Availability

The datasets generated and/or analyzed during the current study are not publicly available due to national security requirements but are available from the corresponding author upon request.

## Abbreviations

Er, Cr:YSGG: Erbium, chromium-doped: yttrium, scandium, gallium, and garnet
mm: Millimeter
J/cm^2^: Joules per square centimeter
Hz: Hertz
nm: Nanometer
Vit. C: Vitamin C
Nd: YAG: Neodymium-doped yttrium aluminum garnet
Q-switched: Quality-switched
EMLA: Eutectic mixture of local anesthetics
VAS: Visual analogue scale
SPSS: Statistical package for the social sciences
*X*^2^: Chi-square
H0: Null hypothesis
H1: Alternative hypothesis
ANOVA: Analysis of variance.

## References

[1] Vachiramon V., McMichael A. J. (2012). Approaches to the evaluation of lip hyperpigmentation. *International Journal of Dermatology*.

[2] Feller L., Masilana A., Khammissa R. A., Altini M., Jadwat Y., Lemmer J. (2014). Melanin: the biophysiology of oral melanocytes and physiological oral pigmentation. *Head & Face Medicine*.

[3] Lin J. Y., Fisher D. E. (2007). Melanocyte biology and skin pigmentation. *Nature*.

[4] Dencker L., Lindquist N. G., Tjälve H. (1976). Uptake of 14C-labelled chloroquine and an 125I-labelled chloroquine analogue in some polypeptide hormone producing cell systems. *Medical Biology*.

[5] Birek C., Main J. H. P. (1988). Two cases of oral pigmentation associated with quinidine therapy. *Oral Surgery, Oral Medicine, Oral Pathology*.

[6] Haresaku S., Hanioka T., Tsutsui A., Watanabe T. (2007). Association of lip pigmentation with smoking and gingival melanin pigmentation. *Oral Diseases*.

[7] Duteil L., Cardot-Leccia N., Queille-Roussel C. (2014). Differences in visible light-induced pigmentation according to wavelengths: a clinical and histological study in comparison with UVB exposure. *Pigment Cell & Melanoma Research*.

[8] Sreeja C., Ramakrishnan K., Vijayalakshmi D., Devi M., Aesha I., Vijayabanu B. (2015). Oral pigmentation: a review. *Journal of Pharmacy & Bioallied Sciences*.

[9] Dadzie O. E., Petit A., Alexis A. F. (2013). *Ethnic Dermatology: Principles and Practice*.

[10] Bandyopadhyay D. (2009). Topical treatment of melasma. *Indian Journal of Dermatology*.

[11] Kunachak S., Kunachakr S., Kunachakr S., Leelaudomlipi P., Wongwaisayawan S. (2001). An effective treatment of dark lip by frequency-doubled Q-switched Nd: YAG laser. *Dermatologic Surgery*.

[12] Limpjaroenviriyakul N., Jurairattanaporn N., Kamanamool N., Rojhirunsakool S., Kanokrungsee S., Udompataikul M. (2020). Low-fluence Q-switched Nd:YAG 1064-nm laser versus Q-switched Nd:YAG 532-nm laser in the treatment of hyperpigmented lips: a prospective, randomized, controlled, evaluator-blinded trial. *Lasers in Medical Science*.

[13] Arda O., Göksügür N., Tüzün Y. (2014). Basic histological structure and functions of facial skin. *Clinics in Dermatology*.

[14] Douplik A., Saiko G., Schelkanova I., Tuchin V. V. (2013). The response of tissue to laser light. *Lasers for Medical Applications*.

[15] Iyer V. H., Farista S. (2014). Management of hyperpigmentation of lips with 940 nm diode laser: two case reports. *International Journal of Laser Dentistry*.

[16] Kassem I., Al-Agha M. A. (2016). Lip depigmentation (pinkification) using 810nm diode laser, kassem’s protocol: initial case report. *Smile Dental Journal*.

[17] Alharbi M. A. (2019). Q‐switched double‐frequency Nd:YAG (532 nm) laser is an effective treatment for racial lip pigmentation. *Journal of Cosmetic Dermatology*.

[18] De Almeida Filippo A., Júnior A. S., Torreão P. S., Delorenze L. M., Issa M. C. A., Issa M., Tamura B. (2017). Erbium laser for photorejuvenation. *Lasers, Lights and Other Technologies. Clinical Approaches and Procedures in Cosmetic Dermatology*.

[19] Taher Agha M., Polenik P. (2020). Laser treatment for melanin gingival pigmentations: a comparison study for 3 laser wavelengths 2780, 940, and 445 nm. *International Journal of Dentistry*.

